# Human MFAP1 is a cryptic ortholog of the *Saccharomyces cerevisiae* Spp381 splicing factor

**DOI:** 10.1186/s12862-017-0923-1

**Published:** 2017-03-24

**Authors:** Alexander K. C. Ulrich, Markus C. Wahl

**Affiliations:** 1 Laboratory of Structural Biochemistry, Freie Universität Berlin, Takustr. 6, D-14195 Berlin, Germany; 2 Helmholtz-Zentrum Berlin für Materialien und Energie, Macromolecular Crystallography, Albert-Einstein-Straße 15, D-12489 Berlin, Germany

**Keywords:** Alternative splicing, B-specific proteins, Pre-mRNA splicing, Spliceosome, U4/U6.U5, Tri-snRNP-specific proteins

## Abstract

**Background:**

Pre-mRNA splicing involves the stepwise assembly of a pre-catalytic spliceosome, followed by its catalytic activation, splicing catalysis and disassembly. Formation of the pre-catalytic spliceosomal B complex involves the incorporation of the U4/U6.U5 tri-snRNP and of a group of non-snRNP B-specific proteins. While in *Saccharomyces cerevisiae* the Prp38 and Snu23 proteins are recruited as components of the tri-snRNP, metazoan orthologs of Prp38 and Snu23 associate independently of the tri-snRNP as members of the B-specific proteins. The human spliceosome contains about 80 proteins that lack obvious orthologs in yeast, including most of the B-specific proteins apart from Prp38 and Snu23. Conversely, the tri-snRNP protein Spp381 is one of only five *S. cerevisiae* splicing factors without a known human ortholog.

**Results:**

Using InParanoid, a state-of-the-art method for ortholog inference between pairs of species, and systematic BLAST searches we identified the human B-specific protein MFAP1 as a putative ortholog of the *S. cerevisiae* tri-snRNP protein Spp381. Bioinformatics revealed that MFAP1 and Spp381 share characteristic structural features, including intrinsic disorder, an elongated shape, solvent exposure of most residues and a trend to adopt α-helical structures. In vitro binding studies showed that human MFAP1 and yeast Spp381 bind their respective Prp38 proteins via equivalent interfaces and that they cross-interact with the Prp38 proteins of the respective other species. Furthermore, MFAP1 and Spp381 both form higher-order complexes that additionally include Snu23, suggesting that they are parts of equivalent spliceosomal sub-complexes. Finally, similar to yeast Spp381, human MFAP1 partially rescued a growth defect of the temperature-sensitive mutant yeast strain *prp38-1*.

**Conclusions:**

Human B-specific protein MFAP1 structurally and functionally resembles the yeast tri-snRNP-specific protein Spp381 and thus qualifies as its so far missing ortholog. Our study indicates that the yeast Snu23-Prp38-Spp381 triple complex was evolutionarily reprogrammed from a tri-snRNP-specific module in yeast to the B-specific Snu23-Prp38-MFAP1 module in metazoa, affording higher flexibility in spliceosome assembly and thus, presumably, in splicing regulation.

**Electronic supplementary material:**

The online version of this article (doi:10.1186/s12862-017-0923-1) contains supplementary material, which is available to authorized users.

## Background

Splicing of primary transcripts is an essential step in the expression of many eukaryotic protein-coding genes. During splicing, non-coding intervening sequences (introns) are excised from a precursor (pre-) mRNA and neighboring coding regions (exons) are ligated via two consecutive transesterification reactions [1, 2]. Pre-mRNA splicing is catalyzed by the spliceosome, a highly dynamic, multi-megadalton molecular ribonucleoprotein (RNP) machine that is composed of five small nuclear (sn) RNPs and numerous non-snRNP proteins [3, 4]. For each round of splicing, a spliceosome is assembled in a stepwise fashion. The vast majority of splicing events in *Saccharomyces cerevisiae* (*sc*) is constitutive and involves assembly of a spliceosome across an intron. In a constitutive splice event, U1 and U2 snRNPs recognize the 5’-splice site and branch point sequence of an intron, respectively, forming the A complex. Subsequently, the U4, U5 and U6 snRNPs join as a pre-formed tri-snRNP, giving rise to the pre-catalytic B complex. The B complex is then catalytically activated, yielding first the B^act^ and subsequently the B* complex. The latter can carry out the first transesterification reaction of a splicing event. After step one of splicing, further rearrangements give rise to the C complex, which catalyzes the second transesterification reaction, subsequent to which the spliceosome is disassembled and subunits are recycled [3, 4].

Most primary transcripts in complex, multicellular eukaryotes contain more than one intron and can undergo alternative splicing to yield multiple mature mRNAs originating from the same gene [5]. The lengths of their introns vary considerably and can amount to several hundreds of thousands of nucleotides [6], while their exons are on average much shorter (ca. 120 nucleotides) and more homogeneous in size [7, 8]. Therefore, faithful localization of authentic 5’- and 3’-splice sites in complex, multicellular organisms is thought to occur via the initial assembly of spliceosomal complexes across exons (exon definition), which commits the pre-mRNA to the splicing pathway [9–12]. To allow intron excision, the interactions established during exon definition have to be reorganized to allow a 3’-splice site to be paired with an upstream 5’-splice site. Exon definition may proceed either to a cross-intron A complex [12] or directly to a cross-intron B complex under omission of a cross-intron A stage [13]. Functional pairing of specific splice sites, and thus the decision on a certain splicing pattern, is thought to take place during this conversion of cross-exon to cross-intron spliceosomal complexes [10, 14–16].

In yeast, pre-mRNA processing factor 38 domain containing protein (Prp38) and 23 kDa small nuclear ribonucleoprotein component (Snu23) are integral components of the U4/U6.U5 tri-snRNP [17, 18], stay associated during tri-snRNP integration and B complex formation and leave the spliceosome again during the transition to the B^act^ complex [19]. The human orthologs of Prp38 and Snu23 are also exclusively present at the B complex stage but, in contrast to their yeast orthologs, associate with the pre-catalytic spliceosome independent of the tri-snRNP [20]. This feature they share with seven other non-snRNP proteins, collectively referred to as B-specific proteins. The specific recruitment of B-specific proteins to the spliceosome during cross-exon to cross-intron switching makes them prime candidates as regulators of alternative splicing.

Indeed, for most B-specific proteins there is evidence that they play a role in alternative splicing. In human, the group of B-specific proteins includes Prp38, Snu23, microfibrillar-associated protein 1 (MFAP1), suppressor of mec-8 and unc-52 protein homolog 1 (Smu1), Arg-Glu/Asp-repeat-containing protein (RED), formin-binding protein 21 (FBP21), 38 kDa nuclear protein containing a WW domain (NPW38), NPW38-binding protein (NPW38BP) and ubiquitin-like protein 5 (UBL5) [20]. *Homo sapiens* (*hs*) Prp38 has acquired a veritable, C-terminal arginine-serine-rich (RS) domain, a hallmark of the splicing regulatory serine-arginine-rich (SR) proteins that are largely lacking in yeast [21]. UBL5, also called Hub1, was the first splicing factor that was found to be involved in alterative splicing in human [22] as well as in a rare case of alternative splicing in yeast [23]. In contrast, MFAP1, Smu1, RED, FBP21, NPW38 and NPW38BP lack obvious orthologs in yeast, where alternative splicing is essentially absent. MFAP1, Smu1 and RED have been implicated directly in the modulation of splice site choices in certain pre-mRNAs [24–29].

Presently, the precise functions of B-specific proteins are unknown. In particular, it is not clear to which extent they are important for both constitutive and alternative splicing, whether orthologs of some of these proteins are truly missing in yeast or have evolved so that they are not easily recognized or if yeast harbors other splicing factors that take over the constitutive roles of some of the B-specific proteins.

MFAP1 was first identified as a component of the extracellular matrix [30]. Later, the protein was found in spliceosome preparations [31], was shown to interact with Prp38 in pull-down experiments and to be required for pre-mRNA processing [32]. Interactions between MFAP1 and other B-specific proteins were identified by yeast two-hybrid (Y2H) [21, 33] and in vitro binding studies [21, 34]. Due to its elongated, solvent exposed nature and predicted dense array of short protein binding motifs, MFAP1 was suggested to act as a scaffold or ruler that engages multiple binding partners [34]. Recently, the molecular details of the MFAP1-Prp38 interaction have been revealed by X-ray crystallography [34], representing one of the few structurally characterized interactions between B-specific proteins besides Snu23-Prp38 and Smu1-RED [35].

Here, we investigated whether *S. cerevisiae* contains an ortholog of metazoan MFAP1. Using InParanoid 8 and systematic BLAST searches, multiple sequence alignments, structure-guided interaction studies and a yeast growth assay, we identified the tri-snRNP-specific pre-mRNA-splicing factor, suppressor of *prp38-1* (Spp381), as the so far missing MFAP1 ortholog in *S. cerevisiae*.

## Results

### Identification of MFAP1 orthologs in the eukaryotic tree of life

To investigate when in eukaryotic evolution an MFAP1-coding gene has been acquired, we conducted an ortholog search using the InParanoid 8 orthology analysis tool [36, 37]. The InParanoid methodology [38] uses pairwise BLAST-based all-*versus*-all sequence comparisons to detect orthologs in sets of protein-coding genes from 273 species (covering the major branches of the eukaryotic tree of life and selected prokaryotes), with each gene represented by one protein. To exclude false positive hits that merely arise from co-occurrence of abundant, highly conserved domains, InParanoid uses a strict cut-off criterion of sequence coverage ≥ 50% and BLAST score ≥ 50. Taking into account the presumably low sequence conservation of MFAP1 due to the predicted structural disorder and the absence of folded protein domains [34], we also performed reciprocal best BLAST hit (RBH) searches of MFAP1 proteins against the same 273 sets of protein-coding genes with relaxed cut-off criteria (BLAST score ≥ 30, E-value ≤ 0.01). The RBH method has a relatively high specificity compared to other ortholog detection methods and its specificity is only marginally affected by changes in cut-off values [39].

**Fig. 1:**
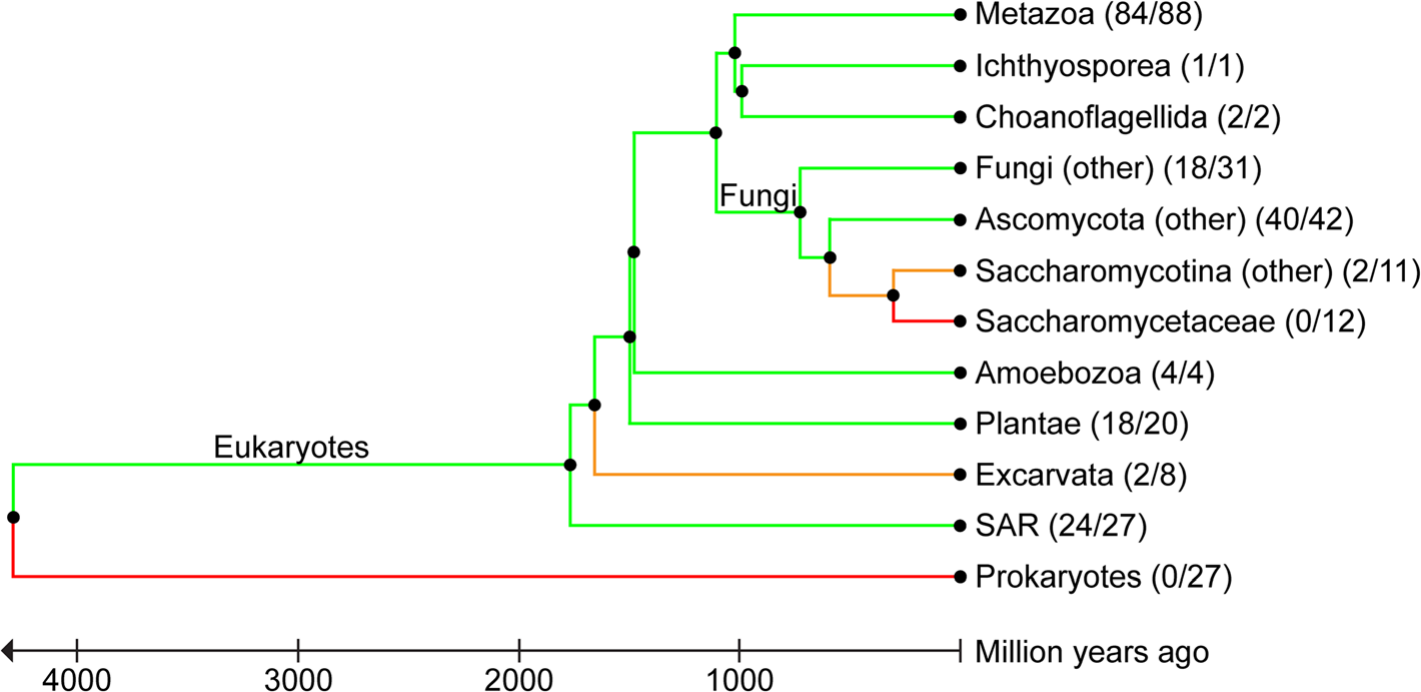
Results summary of *hs*MFAP1 ortholog searches with InParanoid 8. The protein sequence of *hs*MFAP1 (UniProt ID: P55081) was used to search the InParanoid 8 [37] ortholog database and used as templates in BLAST searches against the 273 species (246 eukaryotes plus 27 prokaryotes) covered by the InParanoid 8 program. The phylogenetic tree is based on the divergence times of the taxonomic groups obtained from timetree.org [40, 41]. The number of identified MFAP1 orthologs and the total number of analyzed species in respective taxonomic group is given in brackets. The *branch color* indicates the fraction of the analyzed species that contain an *hs*MFAP1 ortholog; green > 50%, orange > 0% and < 50%, red 0%. See Additional file 1 for detailed results and Additional file 2 for UniProt IDs of identified orthologs

The combined results of both analyses are presented in Fig. 1 and Additional files 1 and 2. An ortholog hit was classified as a high-confidence hit if identified by both methods (black boxes in Additional file 1). Hits delivered by one method alone were classified as medium confidence hits (grey boxes in Additional file 1). The case that the two methods identified two non-identical proteins as orthologous to the query did not occur. Our results show that *hs*MFAP1 proteins are widely distributed in *Metazoa* (95.5% or 84/88 of analyzed species), *Ichthyosporea* (100%, 1/1), *Choanoflagellida* (100%, 2/2), *Amoebozoa* (100%, 4/4), *Plantae* (90%, 18/20), SAR (Stramenopiles, Alveolates, Rhizaria; 88.9%, 24/27), *Fungi* (without Ascomycota; 58.1%, 18/31) and *Ascomycota* (without *Saccharomycotina*; 95.2%, 40/42), but only sporadically present in *Excavata* (25%, 2/8) and virtually absent in *Saccharomycotina* (without *Saccharomycetaceae*; 18.2%, 2/11) and in *Saccharomycetaceae* (0%, 0/12). As expected, MFAP1 was not present in prokaryotes (0%, 0/27) (Fig. 1). MFAP1 seems to be specifically absent in *Saccharomycotina*, as it is present in species that branched off from the human lineage much earlier in evolution than fungi (*Amoebozoa* ca. 1.5 billion years, *Plantae* ca. 1.5 billion years, *Excarvates* ca. 1.7 billion years, SAR ca. 1.8 billion years, fungi ca. 1.1 billion years; estimates obtained from timetree.org [40, 41]) but also in many closely related *Ascomycota* species. In *Saccharomycotina*, MFAP1 was only found in *Yarrowia lipolytica* (*yl*, UniProt ID: Q6CA21) and *Wickerhamomyces ciferrii* (*wc*, UniProt ID: K0KNQ2). Since the latter species is more closely related to *Saccharomycetaceae*, according to divergence time estimations by TimeTree.org ([40, 41]; *wc*: 212 MYA*, yl*: 332 MYA), we performed an additional InParanoid ortholog search with *wc*MFAP1 as a query. We again identified orthologs in many metazoan (78.4%, 69/88), fungal (excluding *Ascomycota*) (48.4%, 15/31) and *Ascomycota* (excluding *Saccharomycotina*) species (81.0%, 34/42). Hits identified in a species with both queries (*hs*MFAP1 and *wc*MFAP1) consistently resulted in the same protein. In addition, using *wc*MFAP1 as a seed, we detected MFAP1 orthologs in all *Saccharomycotina* with the exception of *Saccharomycetaceae* (Additional files 1 and 2). These results indicate that MFAP1 orthologs are present in all major branches of the eukaryotic tree of life but appear to be absent in *Saccharomyces cerevisiae* and its close relatives (*Saccharomycetaceae*).

### Stepwise BLAST searches focused on the fungal kingdom identify Spp381 as a potential MFAP1 ortholog in *Saccharomycetaceae*

**Fig. 2:**
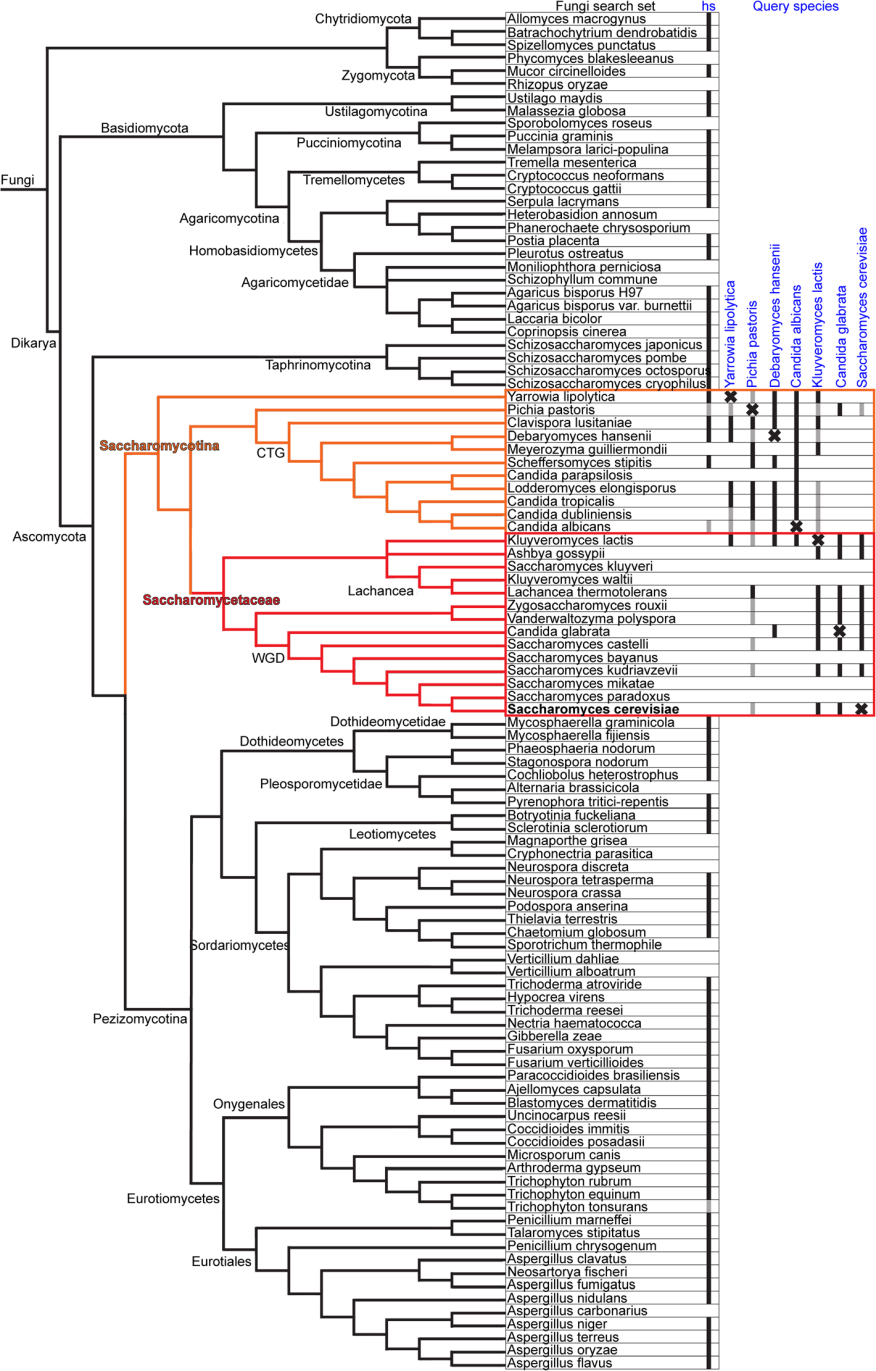
Results of MFAP1 ortholog searches focused on the fungal kingdom. The protein sequence of *Homo sapiens* MFAP1 (UniProt ID: P55081) (hs) was used as template in BLAST searches against 103 fungi that represent the fungal tree of life as published by Medina et al. [42]. Seven MFAP1 orthologs identified in the *Saccharomycotina* subphylum, *i.e.* MFAP1 orthologs of *Y. lipolytica* (UniProt ID: Q6CA21), *P. pastoris* (UniProt ID: A0A1B2J9D1), *D. hansenii* (UniProt ID: Q6BII8), *C. albicans* (UniProt ID: C4YG44), *K. lactis* (UniProt ID: Q6CJ60), *C. glabrata* (UniProt ID: Q6FU95) and *S. cerevisiae* Spp381 (UniProt ID: P38282), were then used as query sequences in further individual BLAST searches against the 25 *Saccharomycotina* species, including 14 *Saccharomycetaceae* species, that are part of the 103 fungal species. The identification of an ortholog within a species is indicated by *black* boxes (high confidence) or *grey* boxes (medium confidence). The fraction of the tree comprising Non-*Saccharomycetaceae Saccharomycotina* nodes is colored in *orange*; the tree fraction comprising *Saccharomycetaceae* nodes is colored in *red*. See Additional file 2 for raw data of BLAST searches

To investigate whether *Saccharomycetaceae* have lost the *mfap1* gene or contain a highly diverged *mfap1* gene, we performed an MFAP1 ortholog search focused on the fungal kingdom with relaxed stringency. The results are summarized in Fig. 2, the raw data are presented in Additional file 3. For this purpose we performed BLAST searches with *hs*MFAP1 as a query against the proteomes of 103 fungal species that represent the fungal tree of life as published by Medina et al. [42]. This tree represents a consensus phylogeny combining three independent phylogenomic approaches (concatenated alignment, single- and multigene supertrees). Although there is a certain overlap between species selected by InParanoid and by Medina et al. and the total number of fungal species is similar (96 vs. 103), the phylogenetic tree by Medina et al. likely represents more accurate phylogenetic relationships among the fungi. To adapt the search to the low sequence similarity usually found between distant MFAP1 orthologs, we used the BLOSUM45 scoring matrix and selected for hits with BLAST score ≥ 30, E-value ≤ 0.01 and query coverage ≥ 20% (high confidence) or ≥ 10% (medium confidence). In addition, we required all further considered hits to represent the best hits in reverse BLAST searches.

As expected, MFAP1 orthologs were detected in the majority of non-*Saccharomycotina* fungi (81.0%, 64/79), as well as in several non-*Saccharomycetaceae Saccharomycotina* species (54.5%, 6/11), but were specifically absent in *Saccharomycetaceae* (0%, 0/14). We assumed that if MFAP1 orthologs exist in *Saccharomycetaceae*, they would be evolutionary closest to neighboring *Saccharomycotina* species. Thus, we repeated the BLAST search with *hs*MFAP1 orthologs identified in the *Saccharomycotina* species *Yarrowia lipolytica* (*yl*, UniProt ID: Q6CA21), *Pichia pastoris* (*pp*, UniProt ID: A0A1B2J9D1), *Debaryomyces hansenii* (*dh*, UniProt ID: Q6BII8) and *Candida albicans* (*ca*, UniProt ID: C4YG44) against the 25 *Saccharomycotina* species of the Medina et al. fungal tree of life. All four species identified MFAP1 orthologs in the majority of non-*Saccharomycetaceae Saccharomycotina* species (*yl*: 7/11; *pp*: 10/11; *dh*: 9/11; *ca*: 11/11). In addition, all four also identified an MFAP1 ortholog in the *Saccharomycetaceae* organism *Kluyveromyces lactis* (Spp381, UniProt ID: Q6CJ60). Furthermore, *Saccharomycetaceae* MFAP1 orthologs were identified in *Candida glabrata* (UniProt ID: Q6FU95) by *dh*MFAP1 and in *Lachancea thermotolerans* by *pp*MFAP1, besides six medium confidence hits (query coverage 10–20%) by *pp*MFAP1. We next selected *K. lactis* and *C. glabrata* MFAP1 orthologs as queries. Both queries identified orthologs in the same nine of 14 *Saccharomycetaceae* species, including *Saccharomyces cerevisiae* (Spp381, UniProt ID: P38282). In addition, the *C. glabrata* protein identified an ortholog in *P. pastoris* and *K. lactis* Spp381 found orthologs in most non-*Saccharomycetaceae Saccharomycotina* species (9/11). Finally, we performed the same analysis with the identified *S. cerevisiae* protein Spp381 as query and found orthologs in the same *Saccharomycetaceae* species (9/14) as with *P. pastoris* and *C. glabrata* in addition to one hit in non-*Saccharomycetaceae Saccharomycotina* (in *P. pastoris*). While the overall low sequence conservation of MFAP1 orthologs is especially pronounced between *Saccharomycetaceae* and neighboring *Saccharomycotina* species, the sequences of the MFAP1 orthologs of *K. lactis* (Spp381) and *P. pastoris* are able to bridge this gap.

To test if Spp381 proteins found in *Saccharomycetaceae* indeed represent a group of MFAP1 orthologs and not a different protein that coexists in MFAP1-containing non-*Saccharomycetceae* species, we used Spp381 from *S. cerevisiae* as a query in our InParanoid-based ortholog search (Fig. 1). *Sc*Spp381 yielded orthologs in *Mycospaerella graminicola*, *Sclerotinia sclerotiorum* (both non-*Saccharomycotina Ascomycota*), *Pichia pastoris* (non-*Saccharomycetaceae Saccharomycotina*) – these proteins are the same as those identified in the initial search with *hs*MFAP1 – and in all twelve *Saccharomycetaceae* species. Thus, we did not find any non-MFAP1 protein as an *sc*Spp381 ortholog. These results show that Spp381 is not closely related, according to the InParanoid cut-off criteria, to any non-MFAP1 protein outside *Saccharomycetaceae*, indicating that Spp381 and MFAP1 do not coexist as different proteins in non-*Saccharomycetaceae* species. However, it is still possible that MFAP1 and Spp381 are highly similar proteins that emerged by convergent evolution and that exist in exactly complementary groups of organisms. It also cannot be excluded that MFAP1 and Spp381 might have emerged from the same ancestral gene by duplication (paralogs) and that a different copy was lost in *Saccharomycetaceae* (*mfap1*) *versus* non-*Saccharomycetaceae* (*spp381*).

### *S. cerevisiae* Spp381 shares physicochemical, biochemical and structural features with *hs*MFAP1

**Fig. 3:**
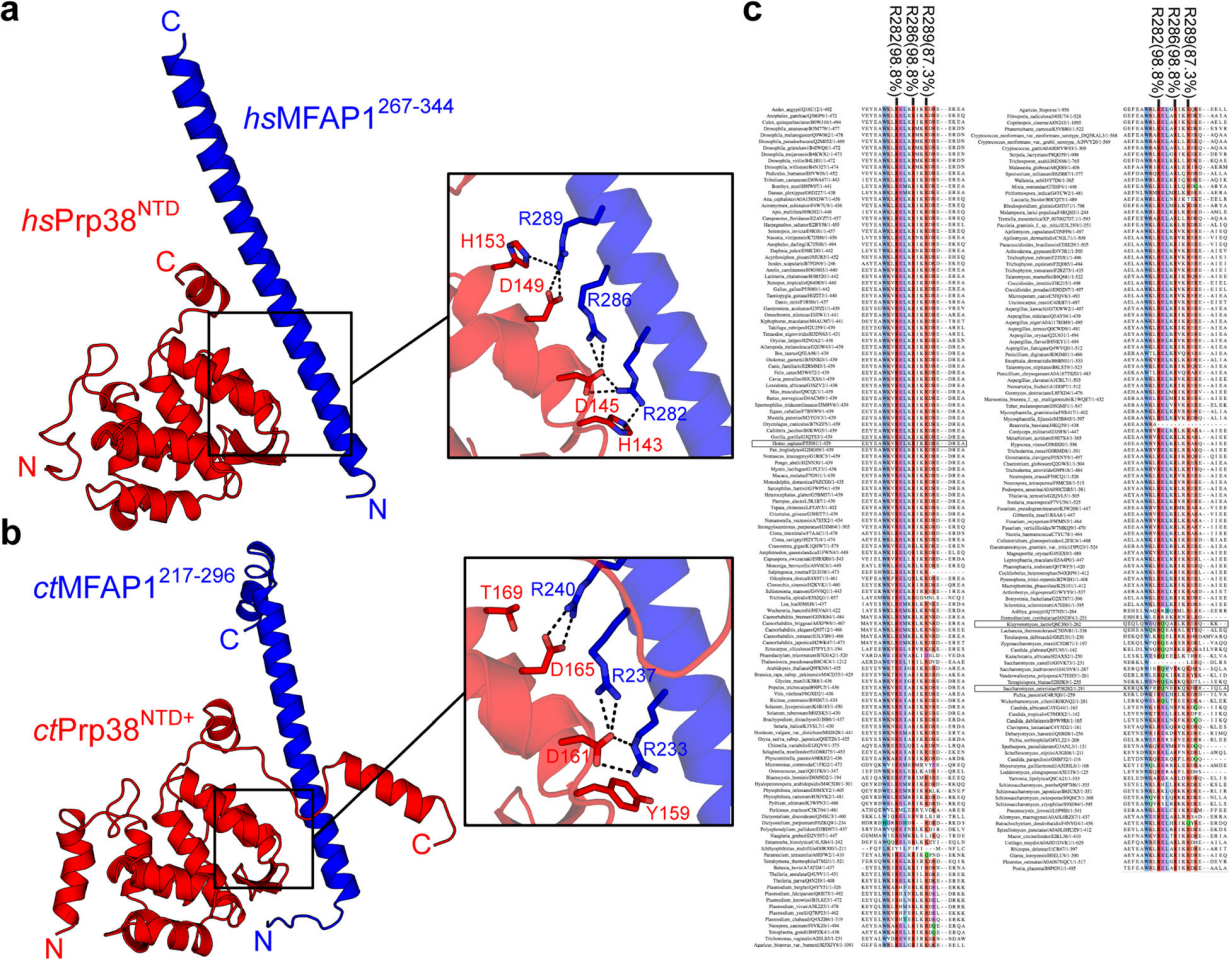
Conservation of the Prp38-MFAP1 interface. **a**, **b** Cartoon representation of (**a**) the heterodimer of *hs*Prp38 (*red*) and *hs*MFAP1 (*blue*) (PDB ID: 5F5S, [34]) or of (**b**) the heterodimer of *hs*Prp38 (*red*) and *hs*MFAP1 (*blue*) (PDB ID: 5F5T, [34]). Key interaction residues are presented as sticks (*right panels*). *Dashed, black lines* indicate hydrogen bonds and salt bridges. **c** Excerpt from a multiple protein sequence alignment of MFAP1 orthologs identified in this study. Key Prp38-interacting residues (R282, R286, R289 according to the *hs*MFAP1 sequence) are marked; percentage identity is given in brackets. In general, residue color intensity indicates level of sequence identity at that specific position; coloring starts at a sequence identity of 30%. *Blue* - conserved hydrophobic residues; *red* – conserved positively charged residues; *purple* – conserved negatively charged residues; *green* – conserved glutamines; *cyan* – conserved histidines. *H. sapiens* MFAP1, *K. lactis* Spp381 and *S. cerevisiae* Spp381 are highlighted by boxes

The rather weak sequence similarity of *Saccharomycetaceae* Spp381 proteins to MFAP1 proteins (e.g. *hs*MFAP1 vs. *sc*Spp381: 13.8% identity, 27.4% similarity; *hs*MFAP1 *vs. kl*Spp381: 14.4% identity, 23.5% similarity) renders an orthology assumption difficult if based on primary sequence data alone. To further test the assumption that MFAP1 and Spp381 proteins are orthologs and not just randomly best-matching proteins, we compared structural and functional data. Intriguingly, *S. cerevisiae* Spp381, like MFAP1, is a known splicing factor [43]. Moreover, *sc*Spp381 had been identified by its ability to suppress defects elicited by the *prp38-1* allele [43], which is associated with impaired spliceosome catalytic activation [44, 45], and its C-terminal half has been shown to directly interact with *sc*Prp38 in Y2H assays [43], the ortholog of *hs*Prp38 that interacts with *hs*MFAP1. Recent crystal structures of the *hs*Prp38-*hs*MFAP1 complex (PDB ID: ID: 5F5S, Fig. 3a) and of the structurally highly similar Prp38-MFAP1 complex from the thermophilic fungus *Chaetomium thermophilum* (*ct*; PDB ID: 5F5T, Fig. 3b) together with binding studies of arginine-to-alanine mutants of the first and second arginine (R282, R286), which were sufficient to disrupt Prp38-MFAP1, revealed a RxxxRxxR motif as a key Prp38-binding element of MFAP1 [34]. Strikingly, the C-terminal halves of *K. lactis* and *S. cerevisiae* Spp381 contain an identical or slightly modified motif, RxxxRxxR and RxxxRxxK, respectively (Fig. 3c and Additional file 4). Among MFAP1 orthologs identified in this study, the first and second arginine residues are conserved in 98.8% and the third arginine in 87.3% of the cases, suggesting that most identified MFAP1 orthologs interact with Prp38 as well.

**Fig. 4:**
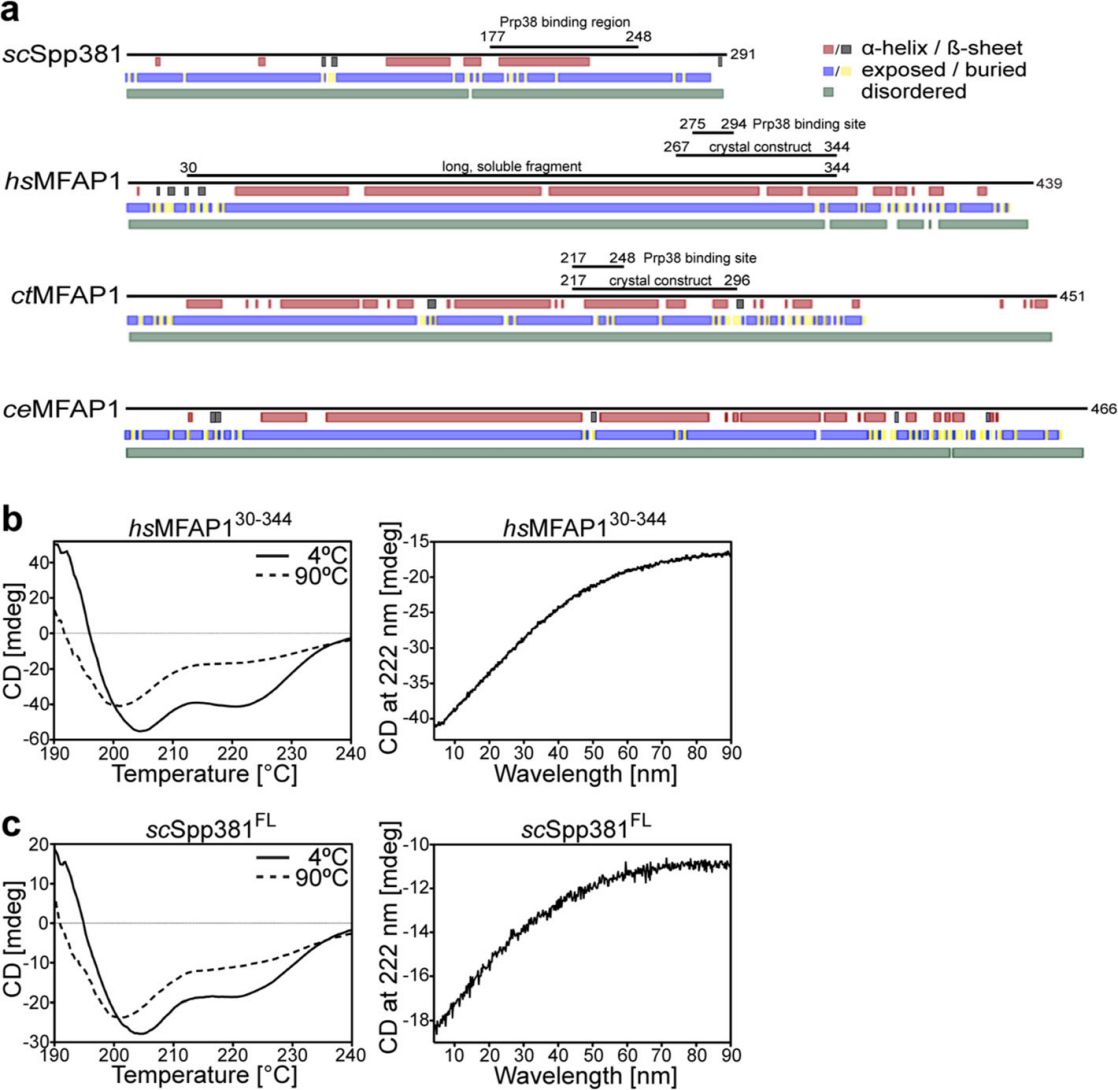
Domain organization and CD spectra of MFAP1 and Spp381. **a** Secondary structure prediction and domain organization of Spp381 from *S. cerevisiae* and of MFAP1 from *H. sapiens*, *C. thermophilum* and *C. elegans. Red/gray bars* – predicted α-helices/ß-strands; *blue/yellow bars* – predicted solvent exposed/buried regions; *green bars* – regions of predicted structural disorder. **b**, **c** CD spectra of *hs*MFAP1 and *sc*Spp381. CD spectra (λ = 190–240 nm) at 4 °C (*solid lines*) and 90 °C (*dashed lines*) and CD melting curves (4–90 °C) at 222 nm of (**b**) *hs*MFAP1^30-344^ and of (**c**) full-length *sc*Spp381

*Sc*Spp381, *hs*MFAP1 and the two previously known MFAP1 orthologs *ct*MFAP1 [34] and *C. elegans* (*ce*) MFAP1 [25] also share a number of physicochemical and biochemical properties, *i.e.* (1) a high fraction of charged residues (*sc*Spp381 39.2%; *hs*MFAP1 46.5%, *ct*MFAP1 49.9%, *ce*MFAP1 50.9%; UniProt average 23.6%); (2) a low isoelectric point (*sc*Spp381 5.4; *hs*MFAP1 5.0, *ct*MFAP1 6.7, *ce*MFAP1 4.9); (3) an increased apparent molecular mass on SDS-PAGE (35 kDa *sc*Spp381 running at ca. 50 kDa; 52 kDa *hs*MFAP1, 52 kDa *ct*MFAP1 and 56 kDa *ce*MFAP1 running at ca. 75 kDa); and (4) PEST elements, which are expected to reduce the half-lives of the proteins [46], with PEST-scores > 16 (*sc*Spp381 residues 56–95, PEST-score +29.8; *hs*MFAP1 residues 67–85 and 174–198, PEST-scores +27.5 and +25.1; *ct*MFAP1 residues 21–44, 105–140 and 161–178, PEST-scores +19.1, +35.6 and +18.7; *ce*MFAP1 residues 192–223, PEST-score +35.2 [47]). In a multiple sequence alignment of all identified MFAP1 orthologs, the PEST element of *sc*Spp381 aligns with PEST elements *hs*MFAP1 174–198, *ct*MFAP1 105–140 and *ce*MFAP1 192–223. In addition, the proteins are predicted to share a similar structure in isolation, *i.e.* they are predicted to be intrinsically disordered (*sc*Spp381 99.7%, *hs*MFAP1 97.3%, *ct*MFAP1 100.0%, *ce*MFAP1 98.8%; average human protein 21.6%; average *S. cerevisiae* protein 17.0% [48]), with most residues solvent exposed (*sc*Spp381 90.4%, *hs*MFAP1 86.6%, *ct*MFAP1 66.7%, *ce*MFAP1 84.1%) and with a tendency to form α-helices (*sc*Spp381 32.0%, *hs*MFAP1 72.9%, *ct*MFAP1 47.0%, *ce*MFAP1 62.9%; average globular protein 30% [49]) (Fig. 4a). Structural disorder of *hs*MFAP1 and *sc*Spp381 was also previously predicted in independent studies [50, 51]. Indeed, CD spectroscopy showed that *sc*Spp381, like *hs*MFAP1, has significant α-helical content in isolation, which gradually changes to a more random coil structure upon heating with no sharp transition (Fig. 4b and c), indicative of a lack of a stable tertiary fold. Irrespective of the exact evolutionary relationship of yeast Spp381 and metazoan MFAP1 proteins, the above analyses indicate that both proteins are structurally very similar and share a Prp38-binding motif.

### Interaction studies corroborate similar functions of *sc*Spp381 and *hs*MFAP1

**Fig. 5:**
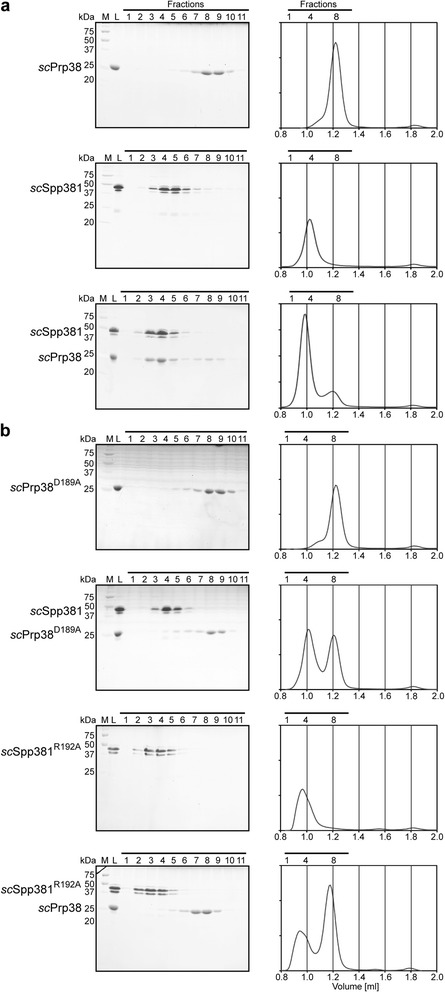
The Prp38-MFAP1/Spp381 interface is conserved between yeast and human. Coomassie Blue-stained SDS-PAGE gels and corresponding chromatograms of analytical gel filtration experiments with full-length *sc*Prp38 and *sc*Spp381. **a** Wild type proteins. **b** Wild type *sc*Spp381 in combination with the *sc*Prp38 D189A variant or wild type *sc*Prp38 in combination with the *sc*Spp381 R192A variant. All experiments were performed with a Superdex 75 3.2/30 column (GE Healthcare). M – marker; L – load; *horizontal black lines* – fractions analyzed by SDS-PAGE

**Fig. 6:**
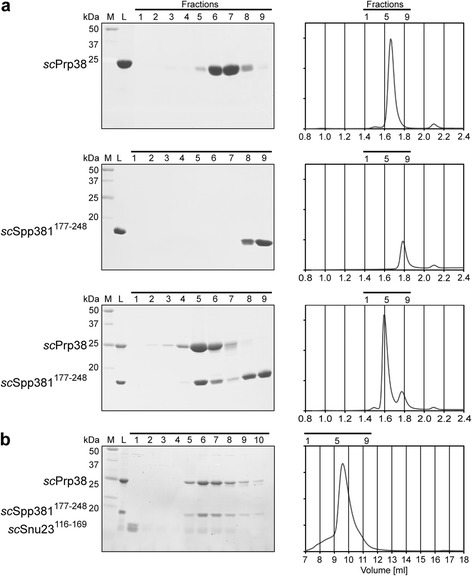
Minimal *sc*Prp38-*sc*Spp381 and *sc*Snu23-*sc*Prp38-*sc*Spp381 complexes resemble analogous MFAP1-based complexes in *H. sapiens* and *C. thermophilum*. Coomassie Blue-stained SDS-PAGE gels and corresponding chromatograms of analytical gel filtration experiments with the indicated *sc*Prp38 and *sc*Spp381 (**a**), or with the indicated *sc*Prp38, *sc*Spp381 and *sc*Snu23 variants (**b**). Experiments in (**a**) were performed with a Superdex 200 increase 3.2/300 column. Experiments in (**b**) were carried out on a Superdex 75 10/300 column (both GE Healthcare). M – marker; L – load; *horizontal black lines* – fractions analyzed by SDS-PAGE

*Sc*Spp381 shares the ability of *hs*MFAP1 to bind Prp38, as shown by Y2H analyses [21, 43]. We confirmed this interaction with isolated, recombinant, wild type *sc*Prp38 and *sc*Spp381 proteins that co-migrated on a gel filtration column (Fig. 5a). To further test if this interaction also uses the same interface as reported in the human and *C. thermophilum* Prp38-MFAP1 complexes [34], we introduced point-mutations into *sc*Prp38 and *sc*Spp381 corresponding to complex-disrupting point-mutations in human Prp38 and MFAP1 (Fig. 3a) and tested interaction of the proteins by analytical gel filtration. Analogous to the Prp38-MFAP1 complexes [34], a D189A mutation in *sc*Prp38 (corresponding to D145A in *hs*Prp38) as well as a R192A mutation in *sc*Spp381 (corresponding to R282A in *hs*MFAP1) led to disruption of the complex (Fig. 5b). Furthermore, *sc*Spp381^177-248^, corresponding to *hs*MFAP1^267-344^, the minimal MFAP1 fragment used for crystallization of the *hs*Prp38-*hs*MFAP1 complex [34], was sufficient to bind *sc*Prp38 (Fig. 6a), further underlining the structural and functional similarities. In addition, we could assemble a trimeric *sc*Snu23^116-169^-*sc*Prp38-*sc*Spp381^177-248^ complex (Fig. 6b), resembling the minimal trimeric Snu23-Prp38-MFAP1 complex in the thermophilic fungus *C. thermophilum*, of which the crystal structure has been solved [34]. These results indicate that *sc*Spp381 and *hs*MFAP1 bind their respective Prp38 partners *via* equivalent interfaces and *via* the same key residues, and that Spp381 is involved in the same trimeric complex as MFAP1.

**Fig. 7:**
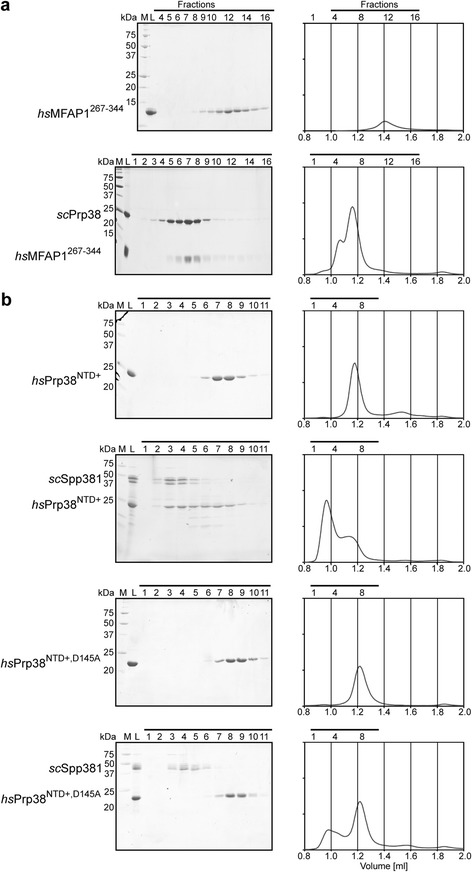
Cross-species interaction studies. Coomassie Blue-stained SDS-PAGE gels and corresponding chromatograms of analytical gel filtration experiments. **a** *Sc*Prp38 binds *hs*MFAP1^267-344^, a minimal *hs*Prp38-interacting fragment. **b** *Hs*Prp38^NTD+^, but not *hs*Prp38^NTD+,D145A^, binds *sc*Spp381. All experiments were performed with a Superdex 75 10/300 column (GE Healthcare). M – marker; L – load; *horizontal black lines* – fractions analyzed by SDS-PAGE

To investigate if the structural similarity between *sc*Spp381 and *hs*MFAP1 is high enough so that they can substitute for each other in Prp38 binding, we performed cross-species interaction studies. Indeed, *sc*Prp38 bound *hs*MFAP1^267-344^ (Fig. 7a) and *hs*Prp38^NTD+^, lacking the complex, multicellular organism-specific RS domain, stably interacted with *sc*Spp381 (Fig. 7b). The latter interaction did not form with the D145A variant of *hs*Prp38^NTD+^ (Fig. 7b). These results show that Spp381 and MFAP1 can substitute for each other in spliceosomal complexes and thus might share a similar interaction network in the spliceosome.

### Human MFAP1 can partially substitute for yeast Spp381 in its function to rescue the conditionally lethal mutant yeast strain *prp38-1*

**Fig. 8:**
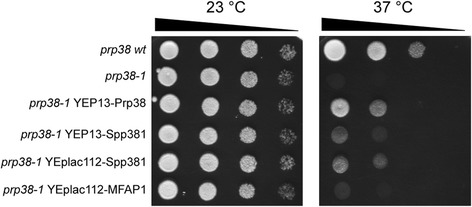
*sc*Spp381 and *hs*MFAP1 partially suppress the growth defect of temperature sensitive yeast strain *prp38-1*. YPD-agar plates were incubated for 3 days at 23 or 37 °C. Each row contains serial dilutions (initial OD_600_: 2.0, 0.2, 0.02, and 0.002) of the indicated yeast strains. Experiments were conducted in triplicates; representative examples are shown

The conditionally lethal yeast strain *prp38-1* produces a mutant version of the Prp38 protein and displays a growth defect at 37 °C [44]. Expression of plasmid-encoded wild type *sc*Prp38 but also of *sc*Spp381 efficiently suppresses this growth defect [43]. To test if *hs*MFAP1 can exploit its capability to bind *sc*Prp38 in a *sc*Spp381-like manner to also functionally substitute for *sc*Spp381 in vivo, we performed yeast growth assays. As expected, all tested *prp38* and *prp38-1* strains grew equally well at 23 °C (Fig. 8, left panel). At 37 °C (Fig. 8, right panel), wild type *prp38* displayed slightly reduced growth compared to 23 °C (row 1). As previously reported, *prp38-1* showed complete growth arrest at 37 °C (row 2). Growth of *prp38-1* at 37 °C was largely restored by transformation with a plasmid encoding wild type *sc*Prp38 (YEp13-2, row 3), partially restored by plasmids encoding *sc*Spp381 (YEp13-7 and YEplac112-7A, rows 4–5) and weakly restored by a plasmid encoding *hs*MFAP1 (YEplac112-MFAP1, row 6). Although expression of plasmid-encoded *hs*MFAP1 did not suppress *prp38-1* as efficiently as over-production of *sc*Spp381, we conclude that *hs*MFAP1 can fulfill certain Prp38-supporting functions of *sc*Spp381 in yeast.

## Discussion

### *Hs*MFAP1 is a cryptic ortholog of the yeast splicing factor Spp381

Proteomics analyses revealed that almost all factors required for constitutive splicing in *S. cerevisiae* are also present in human spliceosomes [4, 19]. Presently, yeast proteins with missing human orthologs include the U1 factors Prp42 and Snu56, the Prp19-associated complex protein Ntc20, the disassembly factor Ntr2 [19] and the U4/U6.U5 tri-snRNP-specific protein Spp381. Compared to yeast, human spliceosomes include ~ 80 additional, predominantly non-snRNP proteins, whose precise functions during splicing are in many cases unclear [4, 19].

Here, we applied the ortholog detection tool InParanoid 8 as well as stepwise BLAST searches to identify MFAP1 as a likely ortholog of the *S. cerevisiae* tri-snRNP-specific protein Spp381. By phyletic profiling we unambiguously identified MFAP1 orthologs in nearly all major branches of the eukaryotic tree of life, including in organisms that split from the common lineage with multicellular eukaryotes about 1.8 billion years ago [40, 41], with the exception of *Saccharomycetaceae*, that separated 1.1 billion years ago [40, 41] (Table 1), where stepwise BLAST searches instead uncovered the Spp381 protein. The evolutionary relationship between MFAP1 and Spp381 was further supported by strong structural similarities between *hs*MFAP1 and *sc*Spp381 that would allow Spp381 to fulfill a role as a flexible scaffolding factor as proposed for MFAP1 [34]. Finally, we presented two key functional indications supporting the assumed evolutionary connection. First, our interaction studies with wild type proteins, single point mutants that failed to interact and cross-species interactions between *hs*Prp38/*sc*Prp38 and *hs*MFAP1/*sc*Spp381, showed that the *sc*Prp38-*sc*Spp381 complex is established *via* a very similar interface to the one observed in the recently structurally characterized *hs*Prp38-*hs*MFAP1 complex [34]. Although we cannot completely rule out the possibility that *hs*MFAP1 and *sc*Spp381 evolved independently to bind the same surface on Prp38, it is rather unlikely that in this case both interactions would rely on exactly corresponding residues. In addition, MFAP1 and Spp381 both bind Prp38 in the context of a trimeric complex with Snu23, further increasing the likelihood of an evolutionary relationship between MFAP1 and Spp381. Second, *hs*MFAP1, like *sc*Spp381, weakly suppresses the temperature-induced growth defect of yeast strain *prp38-1,* most likely by interacting with and stabilizing the mutated Prp38 protein, suggesting that *hs*MFAP1 can fulfill certain *sc*Spp381 functions in vivo. We acknowledge the possibility that a protein that is evolutionarily unrelated to Spp381 might also be able to bind and stabilize the mutated Prp38 protein in *prp38-1*. However, the ability to rescue this growth defect likely requires a set of specific features, including a specific binding mode to Prp38, certain physicochemical properties and the ability to interact with additional binding partners, that seem be overlapping between *hs*MFAP1 and *sc*Spp381 to a large degree and are unlikely to be shared by unrelated proteins. The reduced level of suppression by *hs*MFAP1 compared to *sc*Spp381 might be explained by a lower expression level of plasmid-encoded *hsmfap1* compared to plasmid-encoded *scspp381* in the *prp38-1* strain context, a potentially tighter interaction of *sc*Prp38-*sc*Spp381 *versus sc*Prp38-*hs*MFAP1 and/or the inability of *hs*MFAP1 to bind one or more binding partners of *sc*Spp381 other than Prp38 in yeast. The latter two possibilities are supported by the nature of the protein-binding sites of MFAP1 and Spp381; they comprise short, peptide motif-like sequences with limited structural restraints [34]. Thus, the binding sites are highly likely, over the course of evolution, to strongly adapt to their diverging interaction partners. This notion is in agreement with the overall low sequence similarity between *sc*Spp381 and *hs*MFAP1.

**Table 1:** Summary of ortholog analyses

Search set^a^	Query species^b^								
		*Saccharomycotina*					*Saccharomycetaceae*		
	*hs*	*wc*	*yl*	*pp*	*dh*	*ca*	*kl*	*cg*	*sc*
Eukaryotes (other)^c^	+/+/o	+/+/o	o/o/o	o/o/o	o/o/o	o/o/o	o/o/o	o/o/o	-/-/o
Fungi (other)^d^	+/+/+	+/+/o	o/o/o	o/o/o	o/o/o	o/o/o	o/o/o	o/o/o	-/-/o
*Ascomycota* (other)^e^	+/+/+	+/+/o	o/o/o	o/o/o	o/o/o	o/o/o	o/o/o	o/o/o	-/+/o
*Saccharomycotina* ^f^	+/+/+	+/+/o	o/o/+	o/o/+	o/o/+	o/o/+	o/o/+	o/o/+	-/+/+
*Saccharomycetaceae*	-/-/-	-/-/o	o/o/+	o/o/+	o/o/+	o/o/+	o/o/+	o/o/+	+/+/+

_/_/_ Search strategy: InParanoid database search/InParanoid BLAST search/fungi-focused BLAST search

+ At least one MFAP1 ortholog was identified in the respective search set

- No MFAP1 ortholog was identified in the respective search set

o Search set was not used in the respective analysis

^a^Taxomeric group that was used as the search set

^b^MFAP1 ortholog of this species was used as a search query

^c^Eukaryotes other than fungi

^d^Fungi other than *Ascomycota*

^e^ *Ascomycota* other than *Saccharomycotina*

^f^ *Saccharomycotina* other than *Saccharomycetaceae*

Taken together, the sequence similarity between MFAP1 and SPP381 does not suffice to delineate their precise evolutionary relationships. Yet, they are structurally and functionally similar to an extent that they can substitute for each other. This suggests that, indeed, both proteins may represent orthologs although other evolutionary scenarios cannot be entirely ruled out.

### Functional characteristics of MFAP1 and Spp381 proteins may allow for high evolutionary rates of sequence divergence

Identification of a common evolutionary origin of proteins by sequence comparisons is increasingly challenging with decreasing sequence conservation. Fast diverging sequences lack the evolutionary pressure commonly associated with the maintenance of a particular 3D fold or of extended interaction surfaces. The human B-specific protein MFAP1 is characterized by a lack of stable tertiary structure, structural flexibility and relatively short, but nevertheless high-affine, protein-protein interaction sites and plays a role as an elongated scaffolding factor that could transmit conformational changes within the spliceosome [34]. These functional characteristics likely allow for a high sequence divergence rate during evolution, in particular in regions of the protein that only require the maintenance of an elongated, flexible structure.

Indeed, the sequence identity between known MFAP1 orthologs is low and even less recognizable for evolutionary distant MFAP1 orthologs identified in our study (Additional file 5). In this context it is not surprising that MFAP1 and *Saccharomycetaceae* Spp381 sequences also exhibit a low sequence identity. More surprising, however, is the low sequence conservation between *Saccharomycotina* and other *Ascomycota* species, between *Saccharomycotina* and *Saccharomycetaceae*, and even between neighboring *Saccharomycetaceae* organisms (Additional file 5).

### Liberation from the tri-snRNP may enable B-specific proteins to perform their functions in a regulated manner

**Fig. 9:**
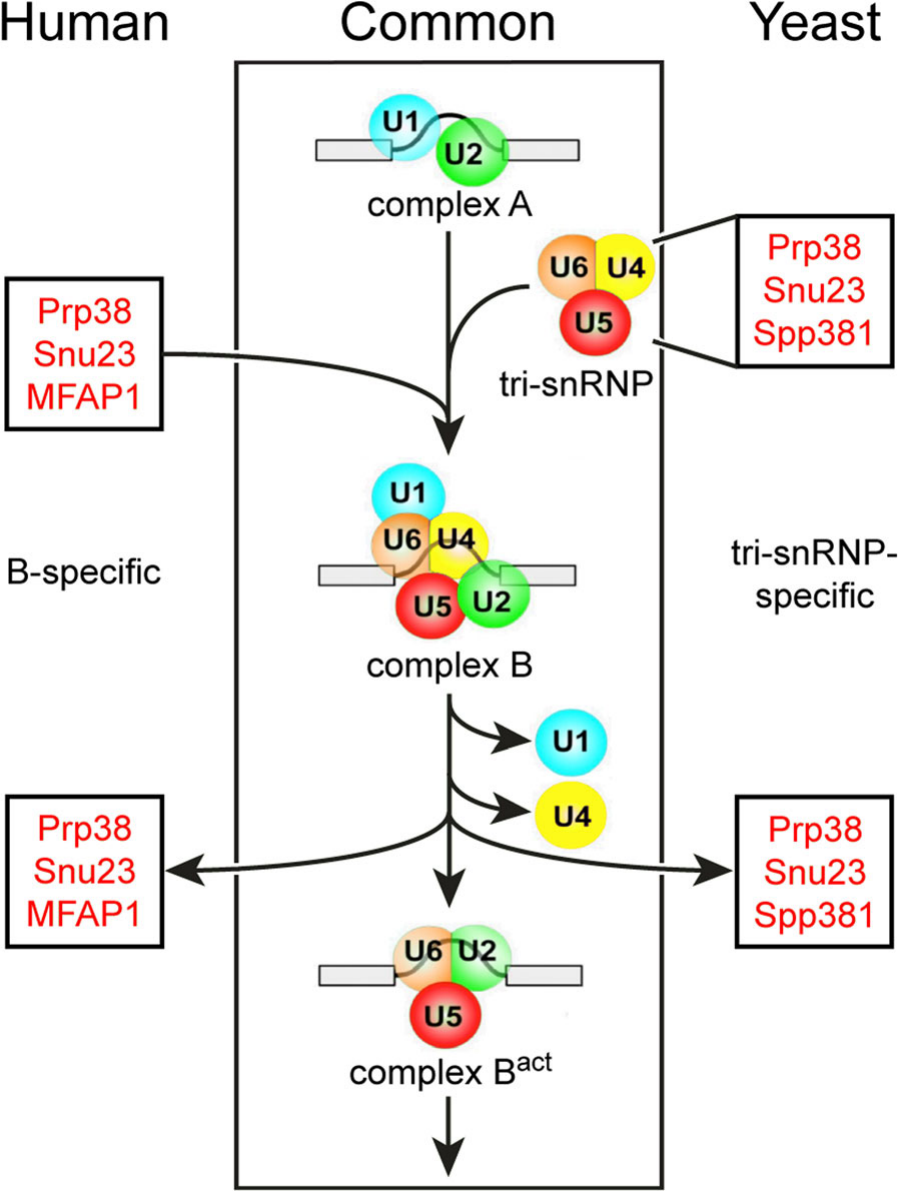
Recruitment of Prp38, Snu23 and MFAP1/Spp381 to the human or yeast spliceosomes. Human Prp38, Snu23, MFAP1 and other B-specific proteins enter the spliceosome at the complex B stage independent of the tri-snRNP. In contrast, yeast Prp38, Snu23 and the *hs*MFAP1 ortholog *sc*Spp381 are tri-snRNP-specific proteins and thus first bind to the tri-snRNP. Subsequently, the tri-snRNP enters the spliceosome to form complex B. In human and yeast, Prp38, Snu23 and MFAP1/Spp381 leave the spliceosome during B to B^act^ complex transition

In addition to the large number of human splicing factors that do not have an obvious conserved counterpart in yeast [4, 19], “reprogramming” of splicing factors Prp38 and Snu23 from stable snRNP components in yeast to non-snRNP proteins in human (Fig. 9) illustrates a lower level of fixed pre-organization of metazoan spliceosomes, even with respect to core splicing factors. In yeast, *sc*Prp38 and *sc*Snu23 are recruited at the same time and with the same efficiency as all other U4/U6.U5 tri-snRNP components to cross-intron spliceosomal A complexes [19]. While their precise roles during spliceosome activation are still unclear, it is obvious that in a situation as encountered in yeast, there is no possibility to regulate, for example, the kinetics of spliceosome activation *via* a more or less efficient recruitment of Prp38 or Snu23 compared to other tri-snRNP components. The situation is decisively different in metazoa, where Prp38 and Snu23 are non-snRNP proteins (Fig. 9) [52]. While they are still recruited at the stage of B complex formation, irrespective of whether the B complex originated from a cross intron A complex [20] or a cross-exon complex [13], their binding could, in principle, be regulated independent of the binding of the U4/U6.U5 tri-snRNP. Thus, while *e.g.* Prp38 most likely can influence the efficiency of catalytic activation also in complex, multicellular eukaryotes [32], the timing of when it unfolds this activity could differ, for example, in two competing alternative splicing situations (which may exhibit different compositions, conformations or spatial distributions of components). Differential binding of Prp38 and Snu23 could thus promote catalytic activation of two competing spliceosomal complexes with a different efficiency and thus influence the relative frequency with which mutually exclusive splice sites are used.

Our findings suggest that a similar functional relationship as between yeast and metazoan Prp38 and Snu23 proteins [21] exists between yeast Spp381 and metazoan MFAP1 proteins. As disruption of the *scspp381* gene leads to severe growth defects and accumulation of unspliced pre-mRNA in vivo [43], *sc*Spp381 is an important, albeit not essential, splicing factor that apparently acts in the same process as *sc*Prp38. We showed that *sc*Spp381 and *hs*MFAP1 exhibit cross-species interactions with the respective Prp38 proteins, suggesting that MFAP1 may be responsible for Spp381-like functions in complex, multicellular eukaryotes. During functional pairing of splice sites after initial cross-intron or cross-exon spliceosome assembly, spliceosomes face the problem of locating and bringing together spliceosomal subunits that are bound at the intron ends and thus may be spatially separated [3]. Elongated proteins that are specifically recruited at this stage, such as *hs*MFAP1 and *sc*Spp381, are well suited to help align and gather spatially separated parts of the spliceosome. They could serve as scaffolds or rulers, e.g. during functional pairing of splice sites, by using limited-length binding epitopes arrayed along their sequence to engage multiple binding partners [34]. However, like *sc*Prp38 and *sc*Snu23, *sc*Spp381 is a stable component of the U4/U6.U5 tri-snRNP [17, 18, 43], while *hs*MFAP1, like *hs*Prp38 and *hs*Snu23, is a non-snRNP B-specific protein [20] (Fig. 9). As in the case of yeast and metazoan Prp38 and Snu23, the tri-snRNP nature of *sc*Spp381 mandates that it is always recruited to spliceosomal B complexes together with other tri-snRNP components, thus rendering its function constitutive. In contrast, the non-snRNP, B-specific MFAP1 protein could be differentially recruited in different, mutually exclusive, splicing situations. Such variable recruitment could influence the relative efficiencies with which competing, alternative splice events are carried out.

## Conclusions

Our study revealed the so far uncharted evolutionary backgrounds of the *H. sapiens* B-specific protein MFAP1 and of the *S. cerevisiae* tri-snRNP protein Spp381. Prior to this work, MFAP1 was thought to exclusively exist in spliceosomes of complex, multicellular organisms. We have shown that an MFAP1 ortholog is present not only in *S. cerevisiae* but also in organisms that separated from the common lineage with complex, multicellular eukaryotes about 1.8 billion years ago. Spp381 was suggested to be one of only five yeast splicing factors without a human ortholog. Its evolutionary connection to MFAP1 reduces this number to four, raising the question if finally all ancient yeast splicing factors turn out to be conserved in complex, multicellular eukaryotes. As exemplified by the present study, identifying evolutionary connections between proteins may point to potential functions as well as potential interaction partners of poorly characterized proteins.

## Methods

### Automated search for orthologs by InParanoid 8

Ortholog searches were conducted using InParanoid 8 [36, 37]. InParanoid 8 is based on sets of protein-coding genes of 273 species, where each gene is represented by one protein. These species include the 66 reference species that the ‘Quest for Orthologs’ community has agreed on using plus 207 additional species with completely sequenced genomes and cover all major branches of the eukaryotic tree of life (246 species) and a representative selection of 27 prokaryotes. The InParanoid methodology [38] uses a pairwise BLAST-based all-*versus*-all sequence comparison to detect orthologs. If candidate sequences are orthologs, they should score higher with each other than with any other sequence in the other organism’s set of protein-coding genes. InParanoid further applies special cluster analysis rules to extract all in-paralogs and exclude all out-paralogs [38]. InParanoid uses a strict cut-off criterion of sequence coverage ≥ 50% and BLAST score ≥ 50. The InParanoid 8 ortholog database [36, 37] provides a user interface to find orthologs inferred by the InParanoid algorithm.

Secondly, we performed RBH searches with different MFAP1 or Spp381 protein sequences against the same sets of protein-coding genes of the 273 species selected by InParanoid using the InParanoid web server [36]. A BLAST hit was considered an ortholog if the BLAST score was ≥ 30 with E-value ≤ 0.01, and if the reverse BLAST search, *i.e.* the BLAST hit was used as query in a BLAST search against the set of protein-coding genes of the original query’s organism, resulted the initial query protein as the best hit. This search aims to identify orthologs that do not survive the strict cut-off criteria used for the InParanoid 8 database [37].

### Manual search for orthologs focused on the fungal kingdom

For an MFAP1 ortholog search among the fungi, we performed individual BLAST searches with *Homo sapiens* MFAP1 (UniProt ID: P55081) as a query against the proteomes of 103 fungal species that represent the fungal tree of life as published by Medina et al. [42]. Seven MFAP1 orthologs identified in the *Saccharomycotina* subphylum, *i.e.* MFAP1 orthologs of *Yarrowia lipolytica* (UniProt ID: Q6CA21), *Pichia pastoris* (UniProt ID: A0A1B2J9D1), *Debaryomyces hansenii* (UniProt ID: Q6BII8), *Candida albicans* (UniProt ID: C4YG44), *Kluyveromyces lactis* (UniProt ID: Q6CJ60), *Candida glabrata* (UniProt ID: Q6FU95) and *Saccharomyces cerevisiae* Spp381 (UniProt ID: P38282), were then used as query sequences in further individual BLAST searches against the 25 *Saccharomycotina* species, including 14 *Saccharomycetaceae* species, that are part of the 103 fungal species. A BLAST hit was considered an ortholog of the query protein if the BLAST score (calculated with the BLOSUM45 scoring matrix) was ≥ 30 with an E-value ≤ 0.01 and query coverage ≥ 20% (high confidence) or ≥ 10% (medium confidence), and if the reverse BLAST search resulted in the initial query protein as the best hit.

### Generation of multiple sequence alignment of MFAP1 orthologs

Multiple sequence alignments of MFAP1 orthologs as shown in Fig. 3c and Additional File 4 were built with the MUSCLE algorithm (version 3.8.31; [53]) and displayed with Jalview (version 14; [54]).

### Pairwise sequence alignment

Sequence identity and sequence similarity values were obtained from pairwise sequence alignments by the EMBOSS Needle tool [55] using a BLOSUM62 scoring matrix.

### Protein sequence analyses

The PredictProtein package [56] was used for secondary structure (REPROFSec), solvent exposure (PROFAcc) and structural disorder (Meta-Disorder) predictions.

### Plasmids for recombinant protein production in *E. coli*

Open reading frames (ORFs) encoding *hs*Prp38 or *hs*MFAP1 were amplified from a human cDNA library and cloned into the pETM11 vector using EMP cloning as described [57]. ORFs encoding *sc*Prp38 and *sc*Spp381 were PCR-amplified from *S. cerevisiae* genomic DNA and cloned into the pETM11 vector using EMP cloning [57]. Truncations and point mutations were introduced by inverse PCR as described [57]. The pETM11 vector guides the production of amino-terminally His_6_-tagged, TEV-cleavable fusion proteins.

### Protein production and purification

Proteins bearing an N-terminal, TEV-cleavable His_6_-tag were produced in *E. coli* Rosetta 2 (DE3) or *E. coli* BL21 (DE3) RIL cells in auto-inducing ZY medium [58] for 24 h at 18 °C. The following steps were performed at 4 °C. Cells were resuspended in solubilization buffer (50 mM sodium phosphate, pH 8.0, 500 mM NaCl, 30 mM imidazole, 5 mM β-mercaptoethanol) and lyzed using an EmulsiFlex-C5 cell homogenizer (Avestin). The soluble fraction was separated from the insoluble fraction by centrifugation for 30 min at 55,900 x g in an Avanti J-26 XP centrifuge (Beckman Coulter). Target proteins were captured on Ni^2+^-NTA resin (GE Healthcare), washed with solubilization buffer and eluted with elution buffer (250 mM imidazole, pH 8.0, 300 mM NaCl, 5 mM β-mercaptoethanol). Tags were cleaved with 1:50 TEV during overnight dialysis against 10 mM sodium phosphate, pH 8.0, 300 mM NaCl, 30 mM imidazole, 5 mM β-mercaptoethanol, and cleaved samples were again passed over Ni^2+^-NTA resin. The flow-through was collected, concentrated, and subjected to size exclusion chromatography (SEC) in SEC buffer (10 mM Tris-HCl, pH 8.0, 300 mM NaCl, 0.1 mM EDTA, 1 mM DTT) using Superdex 75 and Superdex 200 columns (GE Healthcare). Peak fractions were analyzed by SDS-PAGE. Fractions containing the target protein were pooled, concentrated, and shock-frozen in liquid nitrogen.

### Analytical gel filtration chromatography

Proteins (50 μM), alone or with an equimolar amount of binding partner, were incubated in SEC buffer for 30 min at 4 °C. 50 μl of sample were analyzed on Superdex 75 PC 3.2/30 or Superdex 200 Increase 3.2/300 size exclusion columns (GE Healthcare) using an ÄKTAmicro system (GE Healthcare) at 4 °C. The peak fractions were inspected by SDS-PAGE.

### Circular dichroism spectroscopy

Proteins were dialyzed against CD buffer (10 mM sodium phosphate, pH 8.0, 50 mM sodium perchlorate) at 4 °C overnight, and diluted to a final concentration of 4.5 μM (*hs*MFAP1^30-344^) or 5.1 μM (*sc*Spp381^FL^). All spectra were recorded with a Jasco J-810 spectropolarimeter using quartz cuvettes with 0.2 mm path length. Initial CD spectra were collected at wavelengths between 190 and 240 nm at 4 °C. CD melting profiles were then recorded by heating the samples to 90 °C at a rate of 2 °C/min and following the CD signal at 222 nm. Final CD spectra were measured at wavelengths between 190 and 240 nm at 90 °C.

### Yeast strains and yeast plasmids

Yeast strains used in this study are MGD353-46D (*MATα leu2-3,112 trp1-289 ura3-52 his3(-) cyh* ^*r*^ *)* and ts192 (*MATα prp38-1 leu2-3,112 trp1-289 ura3-52 his3(-) cyh* ^*r*^), kindly provided by Brian C. Rymond (University of Kentucky). Yeast plasmids used in this study are YEp13-2 (*prp38, leu2, amp* ^*r*^), YEp13-7 (*spp381, leu2, amp* ^*r*^), YEplac112-7A (*spp381, trp1, amp* ^*r*^), all kindly provided by Brian C. Rymond (University of Kentucky), and YEplac112-MFAP1 (*mfap1, trp1, amp* ^*r*^). YEplac112-MFAP1 was produced by using YEplac112-7A as a template and replacing the *sc*Spp381 coding region with the coding region of *hs*MFAP1 by EMP cloning [57].

### Yeast transformation

For generation of electro-competent *S. cerevisiae* cells, a 50 ml YPD culture was inoculated with overnight culture to an OD_600_ of 0.1 and grown at 30 °C and 250 rpm to an OD_600_ of 1.5–10. Cells were harvested by centrifugation for 10 min at 2,000 × *g* and 4 °C, resuspended in 10 ml YPD, 2 ml 1 M HEPES, pH 8.0, 250 μl 1 M DTT, and incubated for 15 min at 30 °C and 250 rpm. Cells were resuspended in 50 ml of ice-cold milliQ H_2_O and again centrifuged for 10 min at 2,000 × *g*, 4 °C. Subsequently, cells were washed with 2 ml ice-cold 1 M sorbitol and centrifuged for 10 min at 2,000 × *g* and 4 °C. Finally, cells were resuspended in 500 μl ice-cold 1 M sorbitol, aliquoted, and directly used for transformation.

Two microgram plasmid were mixed with 50 μl electro-competent *S. cerevisiae* cells and incubated for 15 min on ice. Subsequent to the electric shock at 1,500 V, 500 μl of ice-cold 1 M sorbitol were added and cells were incubated for 2 h at 30 °C and 250 rpm. For selection of plasmid-containing cells, the cell suspension was plated on minimal medium agar plates lacking leucine (in case of YEplac13 plasmids) or tryptophan (in case of YEplac112 plasmids).

### Yeast growth assay

Yeast strains were grown overnight in liquid minimal medium (6.8 g/l yeast nitrogen base without amino acids, 20 g/l glucose, 40.0 mg/l adenine, 19.2 mg/l uracil, 19.2 mg/l L-arginine, 96.0 mg/l L-aspartic acid, 96.0 mg/l L-glutamic acid, 19.2 mg/l L-histidine, 28.8 mg/l L-lysine, 19.2 mg/l L-methionine, 48.0 mg/l L-phenylalanine, 360.0 mg/l L-serine, 192.0 mg/l L-threonine, 14.4 mg/l L-tyrosine, 144.0 mg/l L-valine, and for YEplac112 plasmid-containing strains 57.6 mg/l L-leucine, for YEp13 plasmid-containing strains 38.4 mg/l L-tryptophan, and for strains without plasmid 57.6 mg/l L-leucine and 38.4 mg/l L-tryptophan) at 30 °C and 250 rpm. Subsequently, cultures were diluted to an OD_600_ of 2.0, 0.2, 0.02, and 0.002. 5 μl of each dilution were spotted on YPD-agar plates and plates were incubated at 23 or 37 °C for 3 days.

## Additional files

Additional file 1: Detailed results of MFAP1 and Spp381 ortholog searches with InParanoid 8. The protein sequence of *Homo sapiens* MFAP1 (UniProt ID: P55081) (hs), the putative *Wickerhamomyces ciferrii* MFAP1 ortholog (UniProt ID: K0KNQ2) (wc), or *Saccharomyces cerevisiae* Spp381 (UniProt ID: P38282) (sc) were used to search the InParanoid 8 [37] ortholog database and used as templates in BLAST searches against the 273 species (246 eukaryotes plus 27 prokaryotes) covered by the InParanoid 8 program. Orthologs found in the InParanoid 8 database and identified in BLAST searches against the 273 InParanoid species are marked by a black box; orthologs either found in the InParanoid 8 database or identified by the InParanoid BLAST search are marked with a grey box; species with no identified ortholog are not marked. Species names are colored according to the taxonomic group they belong to. The phylogenetic tree on the right is based on pairwise species distances derived from shared ortholog content as reported by InParanoid 8 [37]. See Additional file 2 for UniProt IDs of identified orthologs. (TIF 695 kb)

Additional file 2: Excel table of the results of MFAP1 and Spp381 ortholog searches with InParanoid 8. UniProt IDs of orthologs identified by BLAST searches against the 273 species covered by the InParanoid 8 program [37] and by searching the InParanoid 8 ortholog database [37] that are indicated in Fig. 1. Orthologs identified by both methods are highlighted in green; orthologs identified by one method alone are highlighted in yellow; red indicates that no ortholog was identified. (XLSX 45 kb)

Additional file 3: Excel table of the results of MFAP1 ortholog searches focused on the fungal kingdom. Data generated by BLAST searches against 103 fungi species (Fig. 2). UniProt IDs of potential orthologs are highlighted in green if the BLAST score (calculated with the BLOSUM45 scoring matrix) was ≥ 30 with an E-value ≤ 0.01 and query coverage was ≥ 20%, or highlighted in orange if the BLAST score (BLOSUM45 matrix) was ≥ 30 with an E-value ≤ 0.01 and query coverage ≥ 10%. Score - BLAST scores, calculated with the BLOSUM45 scoring matrix; Coverage - percentage of query sequence covered by alignment to the database sequence; E-value - Expect-value: number of hits expected to be seen by chance when searching a database of particular size; Identity - percentage of identical residues of query sequence and BLAST hit within the covered region. (XLSX 118 kb)

Additional file 4: Multiple sequence alignment of yeast MFAP1 orthologs. Multiple sequence alignment of 20 MFAP1 orthologs identified in the analyzed *Saccharomycotina* species. The alignment was built with the MUSCLE algorithm (version 3.8.31; [53]) and displayed with Jalview (version 14; [54]). In general, residue color intensity indicates level of sequence identity at that specific position; coloring starts at a sequence identity of 30%. Blue - conserved hydrophobic residues; red – conserved positively charged residues; purple – conserved negatively charged residues; green – conserved polar residues; cyan – conserved tyrosines or histidines; brown – conserved glycines; yellow – conserved prolines. (TIF 2772 kb)

Additional file 5: Excel table of the results of pairwise sequence identity/similarity analyses. The table presents sequence identity and sequence similarity values for pairs of selected MFAP1 orthologs identified in this study. The sequence identity and similarity values were obtained by the EMBOSS Needle tool [55] using a BLOSUM62 scoring matrix. (XLSX 43 kb)

## Abbreviations

*ca*: *Candida albicans*
CD: Circular dichroism
*ce*: *Caenorhabditis elegans*
*ct*: *Chaetomium thermophilum*
*dh*: *Debaryomyces hansenii*
*hs*: *Homo sapiens*
*kl*: *Kluyveromyces lactis*
MFAP1: Microfibrillar-associated protein 1
*pp*: *Pichia pastoris*
Prp38: pre-mRNA processing factor 38 domain containing protein
RBH: Reciprocal best BLAST hit
RS domain: Arginine-serine-rich domain
*sc*: *Saccharomyces cerevisiae*
SDS-PAGE: Sodium dodecyl sulfate polyacrylamide gel electrophoresis
SEC: Size exclusion chromatography
snRNP: small nuclear ribonucleoprotein
Snu23: 23 kDa small nuclear ribonucleoprotein component
Spp381: pre-mRNA-splicing factor suppressor of *prp38-1*
SR protein: Serine-arginine-rich protein
*wc*: *Wickerhamomyces ciferrii*
Y2H: Yeast two-hybrid
*yl*: *Yarrowia lipolytica*
