# Supplementary figures and images for: Human MFAP1 is a cryptic ortholog of the Saccharomyces cerevisiae Spp381 splicing factor
Source: BMC Evol Biol. 2017 Mar 24;17:91. doi: 10.1186/s12862-017-0923-1 (PMC5364666; doi:10.1186/s12862-017-0923-1)

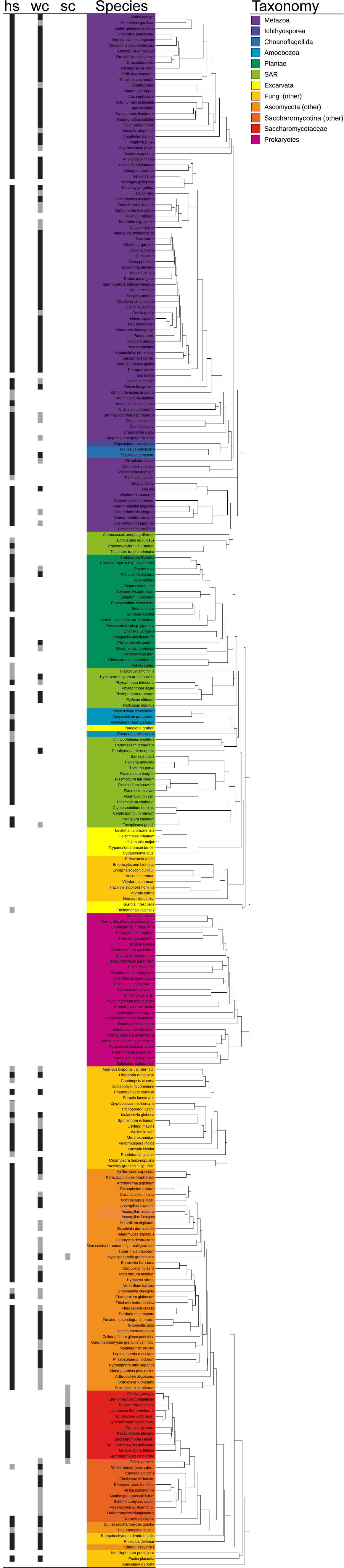

Supplement: Supplementary file 1 — Detailed results of MFAP1 and Spp381 ortholog searches with InParanoid 8. The protein sequence of Homo sapiens MFAP1 (UniProt ID: P55081) (hs), the putative Wickerhamomyces ciferrii MFAP1 ortholog (UniProt ID: K0KNQ2) (wc), or Saccharomyces cerevisiae Spp381 (UniProt ID: P38282) (sc) were used to search the InParanoid 8 [37] ortholog database and used as templates in BLAST searches against the 273 species (246 eukaryotes plus 27 prokaryotes) covered by the InParanoid 8 program. Orthologs found in the InParanoid 8 database and identified in BLAST searches against the 273 InParanoid species are marked by a black box; orthologs either found in the InParanoid 8 database or identified by the InParanoid BLAST search are marked with a grey box; species with no identified ortholog are not marked. Species names are colored according to the taxonomic group they belong to. The phylogenetic tree on the right is based on pairwise species distances derived from shared ortholog content as reported by InParanoid 8 [37]. See Additional file 2 for UniProt IDs of identified orthologs. (TIF 695 kb) [file 12862_2017_923_MOESM1_ESM.tif]

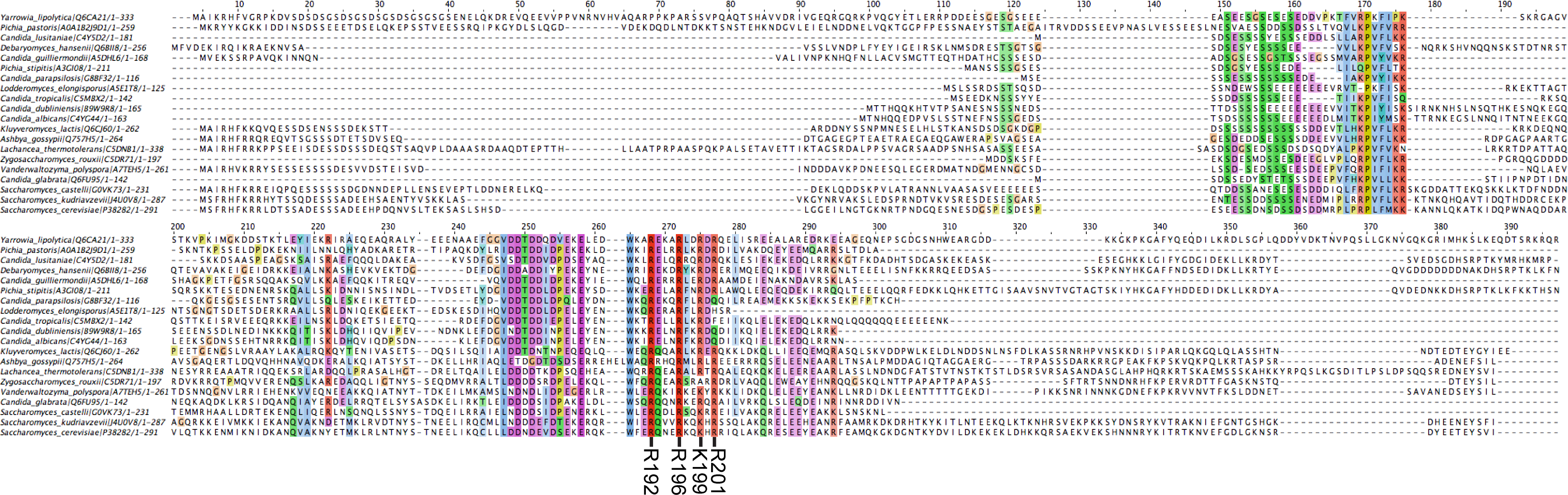

Supplement: Supplementary file 4 — Multiple sequence alignment of yeast MFAP1 orthologs. Multiple sequence alignment of 20 MFAP1 orthologs identified in the analyzed Saccharomycotina species. The alignment was built with the MUSCLE algorithm (version 3.8.31; [53]) and displayed with Jalview (version 14; [54]). In general, residue color intensity indicates level of sequence identity at that specific position; coloring starts at a sequence identity of 30%. Blue - conserved hydrophobic residues; red – conserved positively charged residues; purple – conserved negatively charged residues; green – conserved polar residues; cyan – conserved tyrosines or histidines; brown – conserved glycines; yellow – conserved prolines. (TIF 2772 kb) [file 12862_2017_923_MOESM4_ESM.tif]
